# Renal angiomyolipoma with inferior vena caval thrombus in a 32-year-old male

**DOI:** 10.4103/0970-1591.57927

**Published:** 2009

**Authors:** Matthew W. Christian, Timothy D. Moon

**Affiliations:** Department of Urology, University of Wisconsin, School of Medicine and Public Health, Madison, WI, USA

**Keywords:** Angiomyolipoma, caval thrombus, kidney

## Abstract

Renal angiomyolipoma (AML) rarely presents with evidence of extension into the renal vein, inferior vena cava (IVC) or atrium. We report a case of a renal AML with a tumor thrombus to the IVC in a 32-year-old male. The patient subsequently underwent a right radical nephrectomy with IVC tumor thrombectomy. To our knowledge, there are four published cases of renal AML presenting with tumor thrombus in males. This case report describes the management of the youngest male ever to develop a renal AML with IVC tumor thrombus.

## INTRODUCTION

Renal angiomyolipoma (AML) is a benign neoplasm, consisting of three elements: smooth muscle, adipose tissue and blood vessels. It is predominantly seen in women and its incidence is highest in the fifth or sixth decades of life. It is well associated with tuberous sclerosis, seen in 80% of the patients. Typically, AML is confined to the kidney but, in rare circumstances, tumor can extend beyond the kidney. Surgical treatment is therefore warranted to prevent risk of tumor embolus to the heart or lungs.[1]

## CASE REPORT

**Figure 1:**
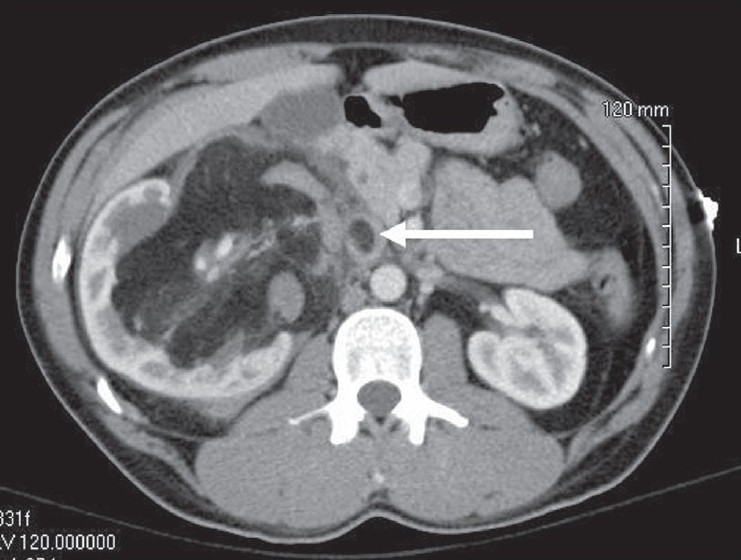
A contrast-enhanced computed tomography scan shows a mass within the right kidney and evidence of tumor thrombus within the inferior vena cava

A 32-year-old male presented with acute and exquisite right upper quadrant pain over a 24-h period. He had no history of tuberous sclerosis. On examination, he experienced tenderness in the right flank and right upper quadrant. His laboratory values were notable for a creatinine of 1.6 mg/dl and a hematocrit of 40%, which was 8 points down from baseline. A computed tomography (CT) scan of the abdomen showed a 13 cm mass arising from the right kidney [Figure 1]. In addition, the IVC contained a tumor thrombus. Unexpectedly, the thrombus was located inferior to the main renal vein. Both the renal mass and the tumor thrombus had negative Hounsfield units consistent with adipose tissue.

Given his uncontrolled pain and falling hematocrit, the patient elected to undergo a right radical nephrectomy. We utilized an anterior subcostal approach and fully mobilized the right kidney. The tumor thrombus was identified inferior to the junction of the main right renal vein and IVC. Here, a lower pole renal vein was seen and surmised to be the source of the thrombus. Throughout the operation, our anesthesiologist closely monitored for any embolus with transesophageal echocardiography (TEE). Next, a vascular surgeon obtained control of the IVC and performed a cavotomy. After the tumor thrombus was removed, the cavotomy was repaired. With the tumor thrombus removed, a right nephrectomy was safely performed. The patient recovered well from surgery and was discharged on post-operative day 7.

**Figure 2:**
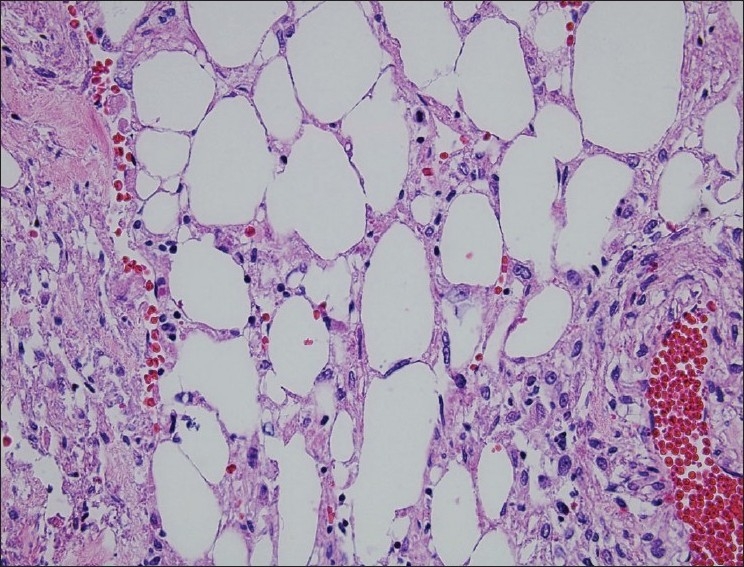
A hematoxylin and eosin-stained section of the renal mass showing smooth muscle, adipose and vascular tissue (400×)

Pathology showed a 14 × 14 × 12 cm renal AML. The tumor thrombus contained the same pathology. There were no atypical or epithelioid features seen on histological examination [Figure 2]. At 1 month after surgery, an abdominal ultrasound showed no evidence of thrombus within the IVC. A CT scan of the abdomen at 2 months post-operatively showed no evidence of recurrence.

## DISCUSSION

Although renal AML is a benign lesion, it has the potential for significant sequela. Retroperitoneal hemorrhage from the AML, known as Wunderlich syndrome, can lead to shock in up to 20% of the patients. In the tuberous sclerosis population, renal failure can occur as they often require multiple procedures to manage their AMLs. Finally, the extension of a renal AML into the renal vein, IVC or atrium is a significant point of morbidity in this disease.[1] There have only been a few case reports of AML with tumor extension in the literature. The majority of these cases are seen in females. To date, only four cases have been described in male patients.[2–5]

Of those men presenting with AML and tumor thrombus, the ages range between 42 and 67, with a mean of 54.5. All these patients had a primary tumor on the right side that extended into the IVC.[2–5] Our case is similar, with a right-sided tumor extending in the IVC. The unique aspect is that he is the youngest, at only 32 years of age. Finally, a comprehensive approach with TEE monitoring for cardiac embolus and collaboration between vascular and urologic surgeons should be considered in similar situations. This effort likely led to an uncomplicated hospital course for our patient.

## References

[1] Bissler JJ, Kingswood JC (2004). Renal angiomyolipomata. Kidney Int.

[2] Rubio-Briones J, Palou Redorta J, Salvador Bayarri J, Miniño Pimentel L, García Penit J, Parada Moreno R (1997). Incidentally detected renal angiomyolipoma with tumour thrombus into the inferior vena cava. Scand J Urol Nephrol.

[3] Cittadini G, Pozzi Mucelli F, Danza FM, Derchi LE, Pozzi Mucelli RS (1996). “Aggressive” renal angiomyolipoma. Acta Radiol.

[4] Bernstein MR, Malkowicz SB, Siegelman ES, Acker M, Tomascewski JE, Wein AJ (1997). Progressive angiomyolipoma with inferior vena cava tumor thrombus. Urology.

[5] Christiano AP, Yang X, Gerber GS (1999). Malignant transformation of renal angiomyolpoma. J Urol.

